# Combined De Novo Transcriptome and Metabolome Analysis of Common Bean Response to *Fusarium oxysporum* f. sp. *phaseoli* Infection

**DOI:** 10.3390/ijms20246278

**Published:** 2019-12-12

**Authors:** Limin Chen, Quancong Wu, Weimin He, Tianjun He, Qianqian Wu, Yeminzi Miao

**Affiliations:** 1 Integrated Plant Protection Center, Lishui Institute of Agricultural and Forestry Sciences, 827 Liyang Stress, Lishui 323000, China; 2 School of Agricultural and Food Science, Zhejiang A&F University, Hangzhou 311300, China

**Keywords:** common bean, *Fusarium oxysproum*, plant–pathogen interaction, transcriptome, metabolome

## Abstract

Molecular changes elicited by common bean (*Phaseolus vulgaris* L.) in response to *Fusarium oxysproum* f. sp. *Phaseoli* (FOP) remain elusive. We studied the changes in root metabolism during common bean–FOP interactions using a combined de novo transcriptome and metabolome approach. Our results demonstrated alterations of transcript levels and metabolite concentrations in common bean roots 24 h post infection as compared to control. The transcriptome and metabolome responses in common bean roots revealed significant changes in structural defense i.e., cell-wall loosening and weakening characterized by hyper accumulation of cell-wall loosening and degradation related transcripts. The levels of pathogenesis related genes were significantly higher upon FOP inoculation. Interestingly, we found the involvement of glycosylphosphatidylinositol- anchored proteins (GPI-APs) in signal transduction in response to FOP infection. Our results confirmed that hormones have strong role in signaling pathways i.e., salicylic acid, jasmonate, and ethylene pathways. FOP induced energy metabolism and nitrogen mobilization in infected common bean roots as compared to control. Importantly, the flavonoid biosynthesis pathway was the most significantly enriched pathway in response to FOP infection as revealed by the combined transcriptome and metabolome analysis. Overall, the observed modulations in the transcriptome and metabolome flux as outcome of several orchestrated molecular events are determinant of host’s role in common bean–FOP interactions.

## 1. Introduction

Fusarium wilt, caused by *Fusarium oxysporum* f. sp. *phaseoli* (FOP), is a destructive soil-borne common bean (*Phaseolus vulgaris* L.) disease. Since its first identification in USA in 1929, this disease has been detected in all bean growing areas such as Africa, East Asia, Europe, Latin America, United Sates, and China [1,2]. Sever fusarium wilt epidemics have been reported in Heilongjiang and other common bean growing areas of China where beans follow vegetables [3]. High moisture, excessive irrigation, or poorly drained fields and a lack of rotation encourage the disease and FOP can persist in the soil indeterminately because of the production of chlamydospores and to the colonization of plant residues including roots of non-susceptible crops cultivated in rotation [3,4]. The majority of studies showed that invasion begins with the hyphal network development around root hairs followed by penetration and colonization of the epidermis and subsequently into the vascular tissues of the root. The colonization in vascular tissues leads it to the stem or the whole plant causing phloem blockage, internal stem discoloration, and total plant wilt. Infected plants display stunting, complete wilting, extensive chlorosis, and necrosis on the leaves [4,5].

Host-*F. oxysporum* pathosystems have been characterized in limited crops i.e., banana (*Musa paradisiaca*) [6,7], melon (*Cucumis melo*) [8,9], chickpea (*Cicer arietinum*) [10,11,12], cotton (*Gossypium hirsutum*) [13], tomato (*Lycopersicon esculentum*) [14], *Arabidopsis* [15,16], *Medicago truncatula* [17]. For each plant species, the respective *Fusarium* pathogens and a variety of defense mechanisms have been observed, including wound responses and hypersensitive reactions as well as many gene expression and metabolic changes. After the infection, plants recognize *Fusarium oxysporum* (FO) attack by understanding endogenous signals originating from the cell-wall through surveillance of cellular intactness. In this regard, many genes such as subtilin-like proteases, leucine rich-repeat proteins, proline-rice glycoproteins, cellulose synthases, and syntaxins are regulated as revealed in melon and banana [6,9,18]. In addition, constitutive enzymatic responses to FO infection appear to be important with changes in glutathione S-transferases, peroxidases, and phenylalanine ammonia lyase enzyme levels and activities being significant upon pathogen attack [19]. Changes also occur in the types and levels of cell-wall proteins, proteinase inhibitors, hydrolytic enzymes, and pathogenesis-related (PR) proteins and phytoalexin biosynthetic enzymes also appear to play important roles in FO defense [19,20,21]. Upregulation of genes involved in shikimate phenylpropanoid-lignin and cellulose synthesis pathways is possibly the reason of resistance in many cultivars where reduced spores are attached and resistance to FO is enhanced [18]. Identification of microbial surface derived molecules i.e., pathogen/microbe-associated molecular patterns (PAMPs/MAMPs) via pattern recognition receptors (PRRs) followed by binding of PAMPs to specific PRRs activates them and sends downstream signals to trigger broad spectrum immunity [22]. In terms of chemical defense, several genes inducing chitinases, xylem proteinases, β-1,3-glucanases thaumatin-like proteins, and peroxidases have been reported to be induced in melon against FOM infection [9]. Hypersensitive response signal molecules such as salicylic acid (SA), jasmonic acid (JA), plant growth regulating hormones, antioxidants, defense metabolites (polyphenols, phenolic acids, and flavonoids), and certain organic acids have been reported to be induced as an active plant defense [9,23]. Certain changes at the metabolic level such as altered activity of genes involved in sugar metabolism (sucrose synthase, invertase, and β-amylase) are also considered a plant response to FO. Sugars offer a dual function in plants as a nutrient as well as a signal to onset of disease, hence, these reactions are important when considering plant defense response [10,24]. The redox status of the intracellular (symplastic) and extracellular (apoplastic) spaces also change with Fusarium wilt infection [25].

In common bean, only a limited number of studies have been conducted to understand the mechanism and pathways involved in response to FOP infection. A recent study using cDNA amplified fragment length polymorphism technique reported transcript-derived fragments functionally characterized as metabolism, signal transduction, protein synthesis and processing, development and cytoskeletal organization, redox reaction, defense and stress response, transport proteins, and gene expression and RNA metabolism related genes/proteins [3]. However proteomic and metabolomics scale responses of common bean against FOP infection is poorly understood.

Unbiased modern high-throughput technologies such as combination of metabolomics and transcriptomics are required to improve our understanding of the plant–fungus interactions in common bean. Such combined approaches have recently resulted in the elucidation of different pathways in plants such as reprogramming of metabolites in chickpea roots in response to FO [12], understanding system responses to brown planthopper and rice stem borer infestation in rice [26,27]. Considering the amount of work done in model plants and other plant species infected with FO, it is important to understand the metabolic changes, transcriptional regulation, or physiological responses of bioactive and signaling compounds during infection of common bean with FOP. We aimed at identifying the differentially expressed genes (DEGs) and metabolites in common bean in response to FOP. The results showed that the transcriptome and metabolome response included structural defense, pathogen recognition receptors, and other components of innate immune system. Hormones played a crucial role in signaling pathways in common bean-FOP pathosystem. The most significantly enriched pathway as per metabolome results and confirmed by transcriptome analysis was the flavonoid biosynthesis pathway. We observed a highly orchestrated response with significant modulation in various metabolic processes. The results described here thus improve our fundamental knowledge of molecular responses to the common bean–FOP interaction and potentially useful in designing strategies against wilt disease in common bean.

## 2. Results

**Figure 1:**
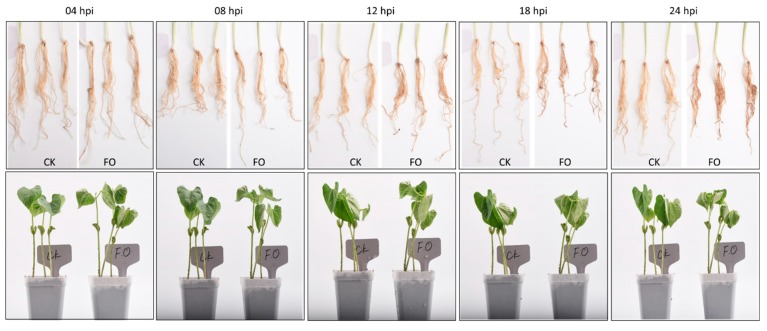
*Fusarium oxysproum* f. sp. *phaseoli* infected (FO) and non-infected (CK) common bean roots and seedlings at 4, 8, 12, 18, and 24 h post infection.

Fusarium wilt progression in common bean (Liyun No. 2) infected with FOP (FO; inoculated) and the control (CK; non-inoculated) was monitored by phenotypic screening at 4, 8, 12, 18, and 24 h post inoculation (hpi). The fusarium infection symptoms i.e., disease incidence index (severity rate 1–5), root length, fresh weight, and root volume were recorded for each time point for nine plants. After 12 hpi, the symptoms started to appear, and disease could be confirmed to a scale of 3. The disease incidence continued to affect roots and shoots. At 24 hpi, the disease incidence reached to a scale of 5 for all nine plants confirming the establishment of the disease. At this time point we confirmed that all treated seedlings at 24 hpi showed significant changes in recorded characteristics (Table S1). The control seedlings in contrast showed normal growth as compared to infected seedlings, remained healthy, and showed no fusarium wilt confirming the successful inoculation of treated plants (Figure 1).

### 2.1. RNA Sequencing and Identification of Differentially Expressed Genes

The transcriptome of six samples of infected and non-infected common bean seedlings were sequenced using the Illumina HiSeq High-throughput sequencing platform. Illumina reads ranging from 43.65 to 48.71 million/sample (on average 46.25 million reads) were obtained from the six samples (Table S2). After filtering low quality reads and adapter sequences, a total of 39.57 Gb clean data was obtained. The clean data of each sample reached 5 Gb, and the Q30 base percentage was 93% or more. The clean data of the six samples of infected and non-infected common bean seedlings were de novo assembled as the reference gene set using the Trinity software and 136,238 unigenes, comprising 195,895,876 bp, were obtained, with a mean length of 1438 bp, a N50 of 2150 bp, and a N90 of 682 bp.

Functional annotation of all the unigenes was conducted, and a total of 80,409 (59.02%), 105,731 (77.61%), 71,638 (52.58%), 7,102,724 (75.4), 61,960 (45.48), 88,302 (64.81%), and 82,599 (60.63%) unigenes were annotated to the Kyoto encyclopedia of genes and genomes (KEGG), non-redundant (NR), Swiss-Prot, Trembl, euKaryotic Ortholog Groups (KOG), Gene ontology (GO), and Pfam database, respectively. Out of all unigenes, 106,777 (78.38%) were annotated in at least one database (Figure S1A). The functional information of homologous sequences in related species showed that the transcript sequences had 68.71%, 9.88%, 2.96%, 2.39%, 2.01%, 1.43%, 1.13%, 0.89%, and 10.59% similarity with *P. vulgaris*, *Quercus suber*, *Vigna angularis*, *Vigna radiate* var. *radiate*, *Cajanus cajan*, *Glycine max*, *Ricinus communis*, *Vigna angularis* var. *angularis*, and other genomes, respectively (Figure S1B). The GO annotation indicated 88,302 unigenes were categorized into 60 functional terms in three categories. Among them, genes associated with metabolic and cellular process in the category ‘biological process’; cell, organelle, and cell part in the category ‘cellular components’; and catalytic activity and transport activity in the category ‘molecular function’, were the most abundant (Figure S1C). The KEGG pathway database was used to analyze intracellular metabolic processes, and 80,409 unigenes were assigned to 144 KEGG pathways (Figure S1D). 

**Figure 2:**
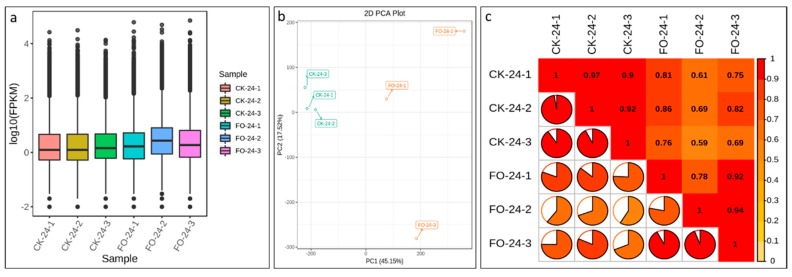
(**a**) Overall distribution of sample gene expression, (**b**) principle component analysis of expressed genes, and (**c**) Pearson correlations between CK-24 and FO-24 replicates.

For the FO-24 samples 84.51–87.26% and for the CK-24 samples 89.17–89.82% reads could be mapped to reference gene set (Table S3). Overall the Fragments Per Kilobase of Transcript per Million fragments mapped (FPKM) for FO-24 seedlings was higher for all three samples as compared to control (Figure 2a). Pearson correlations between FOP inoculated replicates ranged from 0.78 to 0.94 (Figure 2b). The CK replicates clustered together while the FOP inoculated replicates showed variability suggesting that inoculation within the treated plants differed (Figure 2c).

### 2.2. Differential Gene Expression Analysis Related to FOP Infection

**Figure 3:**
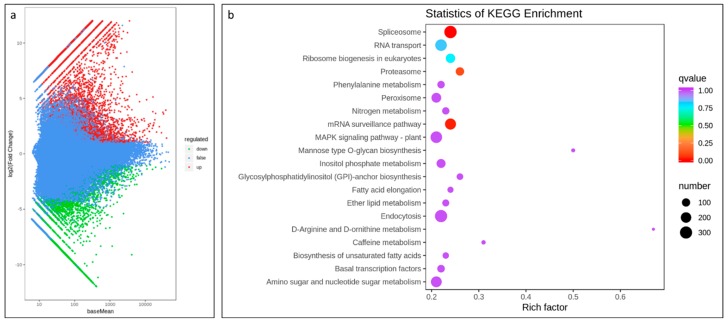
(**a**) Differential gene MA map. The ordinate represents the log2 fold change value; the abscissa represents the average value of gene expression in the two samples; the red dot represents the upregulation of the gene expression, and the green dot represents the downregulation of the expression. Blue indicates no significant difference in gene expression. (**b**) Kyoto encyclopedia of genes and genomes (KEGG) enrichment scatter plot. The ordinate represents the KEGG pathway. The abscissa represents the Rich factor. The larger the Rich factor, the greater the enrichment. The larger the point, the greater the number of differential genes enriched in the pathway. The redder the color of the dots, the more significant the enrichment.

The screening conditions for the differentially expressed genes (DEG) were |log 2 Fold Change| ≥ 1, and False discovery rate (FDR) < 0.05. A total of 22,040 unigenes were expressed differentially with 8269 downregulated and 13,771 upregulated (Figure 3a).

Of the DEGs, 2780 downregulated genes were exclusively expressed in CK-24 while, 8231 upregulated DEGs were exclusively expressed in FO-24. These results show that the transcriptional changes are intense in FO infected common bean seedlings at 24 hpi. We further performed KEGG analysis to look at the key biological pathways involved in response to infection of FOP at 24 hpi. Spliceosome was the significantly enriched pathway with highest ratio of the number of differential genes annotated to this pathway to the number of annotated differential genes i.e., 314 out of 7204 genes in response to FOP infection 24 hpi. Other pathways such as endocytosis, RNA transport, mitogen-activated protein kinase (MAPK) signaling pathway-plant, mRNA surveillance pathway, amino sugar and nucleotide sugar metabolism pathway, and peroxisomes were significantly enriched (Figure 3b).

**Figure 4:**
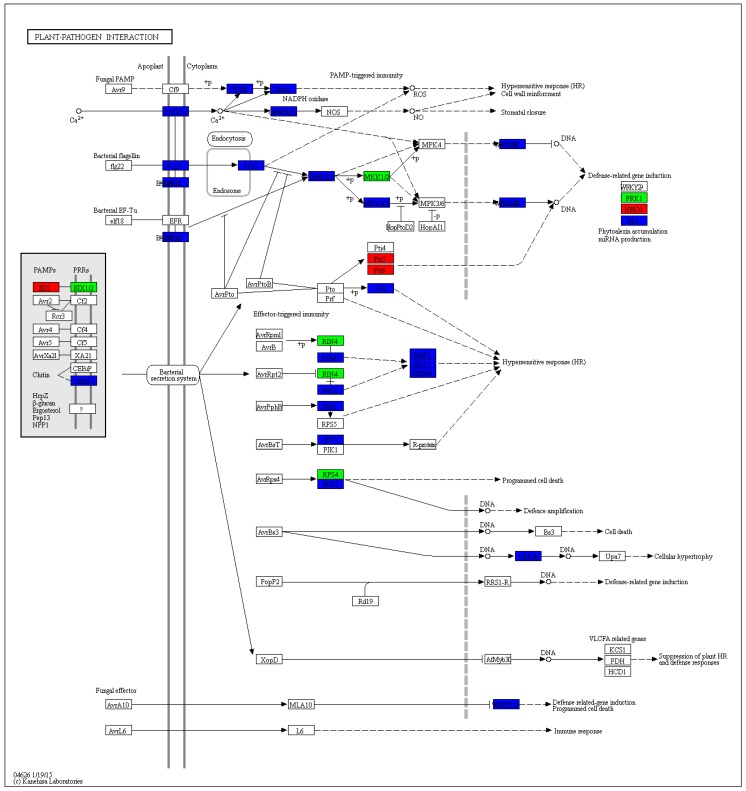
KEGG orthology map (ko04626, plant–pathogen interaction) of common bean-*Fusarium oxysproum* f. sp. *Phaseoli* (FOP) pathosystem. For the treatment group, the red box labeled enzyme is associated with the upregulated gene, and the green box labeled enzyme is associated with the downregulated gene. The blue labeled enzyme is related to both upregulation and downregulation. This pathway map is associated with DEGs. The enzymes are all marked with different colors.

KEGG database (http://www.genome.jp/kegg/) was used to perform pathway mapping of the DEGs involved in common bean–FO interactions to facilitate the inspection of the plant gene networks. KEGG analysis revealed that unigenes were significantly enriched in various components involved in pathogen resistance mechanisms or signaling pathways (Figure 4).

To identify the most potential candidate genes related to resistance mechanism in common bean against FOP, we focused subsequent analysis on the 11,950 DEGs with fold change > 2 (Table S4).

#### 2.2.1. Structural Defense

Plants recognize FOP attack for effective defense. Plants perceive an arsenal of endogenous signals originating from their own cell-wall by surveillance of cellular interactions. The plant cell-wall is the first line of defense for plant cells and defines the primary strength of plants to restrict entry of pathogen to cell. We found 21 subtilisin-like protease and 46 DEGs encoding for leucine rich-repeat (LRR) proteins were of the highly expressed proteins in common bean seedlings infected by FOP 24 hpi. Several genes involved in shikimate phenylpropanoid-lignin and cellulose biosynthesis pathways are reported to strengthen the cell-wall in resistant plants in response to FO infection [18]. We observed higher expression of 3-deoxy-d-arabino-heptulosonate-7-phosphate synthase, a lower expression of coumarate-CoA ligase gene which is reported to strengthen the cell-wall, indicating cell-wall weakening in response to FOP infection 24 hpi. A polyphenol oxidase gene had a very high expression in FOP infected plants as compared to the CK. Similarly, other genes such as UDP-glucuronic acid decarboxylase and cellulose synthases were downregulated in FOP infected plants. Other genes for cell-wall reinforcement have been reported to express during FO infection in tomato [9], we also noticed a shift in expression of four proline-rich glycoprotein, four hydroxyproline-rich glycoprotein, and 18 syntaxin genes. In addition, the bean response to FOP infection 24 hpi was characterized by hyper accumulation of transcripts coding for cell-wall degradation i.e., pectate lyases, pectin methylesterase inhibitors (PMEI), pectin methylesterases (PME), and Polygalacturonases (PG) (Table S4).

#### 2.2.2. Signaling

Plants recognize pathogen surface-derived molecules i.e., (PAMP/MAMP) via PRRs. This binding of PAMP to specific PRR activates these receptors and relays the signal downstream to convergent signaling pathways triggering broad-spectrum immunity. A total of 15 pathogenesis-related (PR) genes were differentially expressed. The fragments released in response to disruption of the first line of defense i.e., cell-wall (galacturonic acid-containing fragments) act as signals and mediate defense response by strengthening defensive barriers i.e., Chitin elicitor-binding protein (CEBiP) and chitin elicitor receptor kinase (CERK). In beans infected with FO-24, seven fungal elicitor immediate early-responsive genes showed higher expression as compared to CK-24. Further, 46 receptor kinases belonging to different gene families were expressed in FO-24 with a limited or zero expression in CK-24.

Involvement of glycosylphosphatidylinositol-anchored proteins (GPI-Aps) with extracellular ligands such as pathogen molecules as well as other ligands i.e., phytohormones, signaling polypeptides, leads to the phosphorylation of the intracellular kinase domain, which consequently activate cytoplasmic signaling components and switch on the response mechanisms. We also observed that glycosylphosphatidylinositol (GPI)-anchor biosynthesis pathway (K000563) was within significantly enriched pathways (Figure 3b). Apart from this, calmodulin (CaM) related DEGs were also induced suggesting the involvement of the CaM dependent signaling pathway. 

The role of hormones in signaling pathways is well established which involves systematic acquired resistance. It has been suggested that resistance to FOP is mediated by SA, jasmonate (JA), and ethylene (ET) pathways [9,18]. In this regard 24 DEGs encoding for Ankyrin repeat containing protein genes were highly expressed in FO-24 beans. Genes responsive to ET are activated during early infection. We observed that four ET-insensitive protein 2 genes were highly responsive to FOP infection in FO-24 bean seedlings. This suggests that ET responsive genes might also be involved in latter infection stages of FOP and should be investigated further. Levels of core JA-signaling related genes i.e., four ZIM domain containing proteins, 12 TIFY, 26 ethylene-responsive transcription factor genes were highly expressed. Among these ethylene-responsive transcription factor-RAP2-7 and RAP2, AP2-like ethylene-responsive transcription factor, ethylene-responsive transcription factor ERF113, ethylene-responsive transcription factor 1, ERF15 were uniquely expressed in FO-24 bean seedlings. There were nine superoxide dismutases expressed in FO-24 in higher fold change values as compared to CK-24 supporting the notion that JA levels are quite high in infected common bean seedlings. Further, the elevated levels of the transcripts of lipoxygenases, linoleate 13/9 S-lipoxygenases, and allene oxide cyclases suggested higher JA levels. However, the role of JA pathway in susceptibility and tolerance FO is still controversial. Almost a five-fold increase in transcript abundance of a defensin-like protein (*PHAVU_005G071400g*) was observed. Homologs of this gene have been reported in *Arabidopsis* and are induced in response to FO infection. It is known that FO infection activates the transcription of auxin-related genes leading to a higher auxin biosynthesis. Three auxin influx career genes, nine auxin induced proteins, and one auxin responsive protein had higher expression in FO-24 seedlings as compared to CK-24. We observed the activation of the MAPK cascade. This pathway was also found to be significantly enriched (Figure 3b).

### 2.3. Validation of DEGs by qRT-PCR 

**Figure 5:**
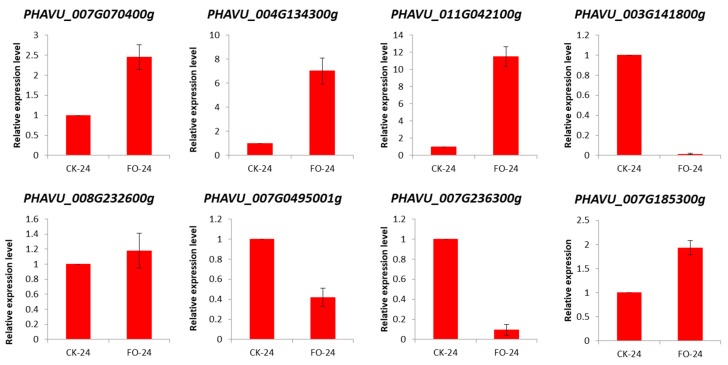
qRT-PCR validation of the selected common bean differentially expressed genes (DEGs) in control (CK-24) and FOP infected plants (FO-24) 24 h post infection.

We validated the expression profiles of eight common bean genes of particular interest (Figure 5). The *Actin* gene was used as an internal control to standardize the data, and the amount of target gene transcript was normalized compared to the constitutive abundance of common bean *Actin* gene [3,28]. Among the common bean DEGs analyzed, five genes were upregulated in FO-24 as compared to CK-24 i.e., *PHAVU_007G070400g*, *PHAVU_004G134300g*, *PHAVU_011G042100g*, *PHAVU_008G232600g*, and *PHAVU_007G185300g*. All these upregulated genes were characterized by similar trend in transcript accumulation at tested hpi supporting the RNA-Seq data. The other three genes i.e., *PHAVU_003G141800g*, *PHAVU_007G0495001g*, and *PHAVU_007G236300g* were downregulated in response to FOP infection in FO-24 seedlings confirming the reliability of our RNA-Seq data. The three downregulated genes are cell wall related, a serine/threonine kinase activity related gene, and an Ankyrin repeat containing gene, respectively.

### 2.4. Metabolite Profile

A combination of UPLC-MS/MS detection platform, self-built database, and multivariate statistical analysis was used to study the differences in metabolome between CK-24 and FO-24. It offers a platform to detect a great diversity of metabolites as previously reported in tomato [29], *Prunus mira* [30], and hulless barley [31,32,33]. In total, 754 metabolites were successfully detected in both sample types (Table S5). The diverse set of detected molecules could be roughly grouped into 23 major classes, predominantly organic acids and derivatives, amino acids and their derivatives, nucleotides and their derivatives, lipids, phenylpropanoids, and flavones. Collectively, phenolics were the major components of metabolome (flavanone, flavone, flavonoid, flavonol, isoflavone, polyphenol, anthocyanins, proanthocyanidins, phenolamides, phenylpropanoids) accounting for 1/3 of the total metabolites detected (Table S5). Moreover, since most of the identified metabolites in this study have not yet been reported in the *Phaseolus vulgaris* metabolic network, our work offers prospects for new bioactive compound discovery.

**Figure 6:**
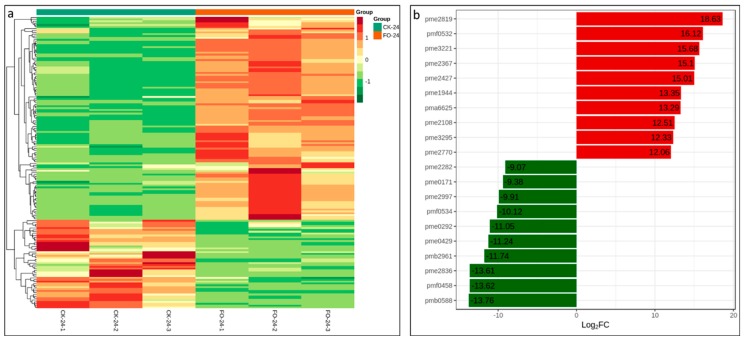
(**a**) Heatmap hierarchical clustering of differentially expressed metabolites. Hierarchical trees were drawn based on differentially accumulated metabolites in CK-24 and FO-24. (**b**) Top 10 differentially accumulated metabolites in CK-24 and FO-24.

We compared the quantitative metabolic profiles between CK-24 and FO-24 roots in order to identify the compounds that differentially accumulated after infection. A series of pairwise OPLS-DA were applied to maximize the discrimination between experimental samples and to focus on metabolic variations significantly contributing to the resulting classifications. The differences between the control and infected groups in the OPLS-DA suggested that significant biochemical perturbation occurred in these samples (Figure S2). All significantly differentially expressed metabolites (fold change ≥ 2 or ≤ 0.5), with the variable importance in the projection (VIP ≥ 1.0) between FO-24 and CK-24 roots are listed in Table S6 and Figure 6a. In total, 158 metabolites were differentially expressed, with 110 upregulated and 48 downregulated.

We found that most of the significantly altered metabolites between FO-24 and CK-24 are phenolic compounds (Table S6). The top 10 most differentially expressed metabolites are listed in Figure 6b. Among them, the upregulated metabolites include All-trans-13,14-dihydroretinol, Phillyroside, Isoeugenol, Quinone, N-Acetyl-L-tyrosine, D-Mannitol, E-3,4,5’-Trihydroxy-3’-glucopyranosylstilbene, L-Carnitine, Prunetin, and L-Cystathionine. Similarly, the top 10 most downregulated metabolites include Luteolin 3’,7-di-O-glucoside, 6-Gingerol, 5-Hydroxytryptophol, Peonidin O-malonylhexoside, 3,4,5-Trimethoxycinnamic acid, (3,4-Dimethoxyphenyl) acetic acid, Hinokitiol, 4-Hydroxycoumarin, N-Isovaleroylglycine, and Guanosine monophosphate.

**Figure 7:**
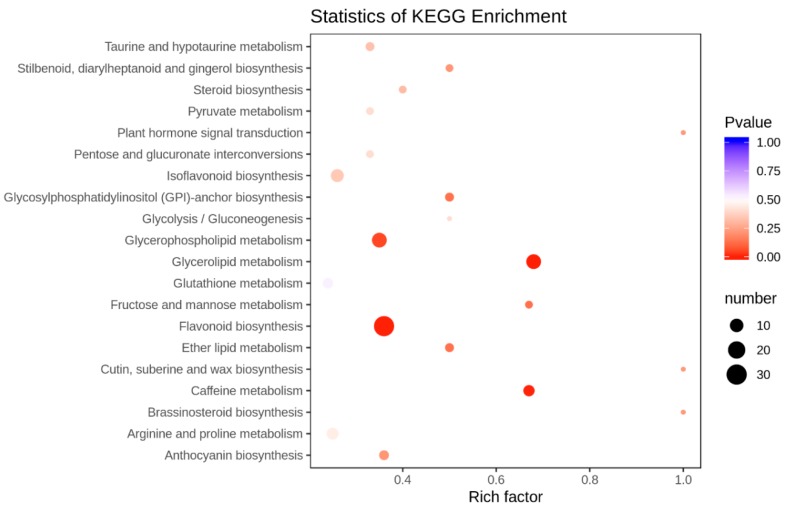
Differential metabolite KEGG enrichment. The larger value indicates that the degree of enrichment is greater. The closer the *p-*value is to 0, the more significant the enrichment is. The size of the points in the graph represents the number of distinct significant metabolites enriched into the corresponding pathway.

Differentially accumulated metabolites were functionally annotated using the KEGG database. It was observed that flavonoid biosynthesis, Glycerolipid metabolism, and Glycerophospholipid metabolism pathways were the most enriched (Figure 7).

## 3. Discussion

### 3.1. Structural Defense in Response to FOP Infection in Common Bean

The present study reports the first combined de novo metabolome and RNA-seq analysis designed to describe the response of common bean infected with FOP. Previously, plant–FOP interaction has been demonstrated in many experiments in different crop plants [6,7,8,9,10,11,12,13,14,15,16]. In common bean it is known that colonization of FOP induces the defense responses in both a constitutive and inducible way. Pathogens are perceived by two different recognition systems that initiate pattern-triggered immunity and effector triggered immunity in order to repel pathogens via induced defense responses [22]. Common bean, like other plants, employs cell-wall as the first barrier and defines primary or basic strength to encounter FOP infection. In this regard, the lower expression of UDP-glucuronic acid decarboxylase and cellulose synthases, coumarate-CoA ligase and hyper accumulation of pectate lyases, PMEIs, PMEs, and PGs indicate that FOP has established itself at 24 hpi and cell-wall weakening in response to FOP infection has started. The higher expression of 3-deoxy-d-arabino-heptulosonate-7-phosphate synthase and polyphenol oxidase highlighted the timely recognition of FOP invasion and induction of the defense system [9,18]. These results confirmed that upon infection and establishment of FOP in common bean root tissues, the FOP secreted enzymes loosen and degrade the cell-wall i.e., pectin, cellulose, and hemicellulose [34]. 

### 3.2. Modulation of Defense Related Proteins in Common Bean

In order to trigger immunity, plants recognize the pathogen surface derived molecules PAMP/MAMP by employing PRRs which in turn activates the receptors and transcends the signals to different pathways and triggers broad spectrum immunity in plants. The PR proteins, among all defense related proteins, are induced and accumulated in response to FOP infection at 24 hpi and are considered an indispensable component of the innate immune system [35]. The 15 differentially expressed PRs in FO-24 suggest that in common bean, the innate immunity system is activated at this stage. Once, the FOP has established itself in bean tissues, the fragments released in response to disruption of the first line of defense i.e., cell-wall (galacturonic acid-containing fragments) act as signals and mediate defense response by strengthening defensive barriers i.e., CEBiP and CERK. In this regard, the high activation of fungal elicitor immediate early-responsive genes in FO-24 as compared to CK-24 confirms that such signals are received by common bean root tissues [18,22,36]. Involvement of GPI-APs with extracellular ligands such as pathogen molecules as well as other ligands i.e., phytohormones, signaling polypeptides, leads to the phosphorylation of the intracellular kinase domain, which consequently activate cytoplasmic signaling components and switch on the response mechanisms. In FO-24 bean roots, the GPI-anchor biosynthesis pathway was one of the significantly enriched pathways both in transcriptome as well metabolome analysis. This confirms that in common bean, GPI-APs is involved in signal transduction in response to FOP infection [37] (Table S3; Figure 3b).

### 3.3. Crucial Role of Hormones in Signaling Pathways in Common Bean-FOP Pathosystem

Role of hormones in signaling pathways is well established which involves systematic acquired resistance. It has been suggested that resistance to FOP is mediated by SA, JA, and ET pathways [9,18]. Previous reports on functional characterization of rice Ankyrin repeat containing proteins confirmed their role in defense against pathogen attack, particularly against *Magnaporthe oryzae* [38]. The contrasting expression of 24 Ankyrin repeat containing genes in FO-24 and CK-24 observed in our transcriptome clearly indicates that bean roots, under FOP infection, employ them as a defense response. Activation of the ET responsive genes at FO-24 is an important observation as previously it was known that some ET responsive proteins such as ET-insensitive protein-2 genes are involved in early infection in banana [18]. Hence their activation/expression at 24 hpi suggests that these genes might be involved in latter FOP infection stages and should be investigated further. The unique expression of JA-signaling related genes in FO-24 seedlings clearly indicates that hormone signaling pathways are involved defense responses in common bean against FOP infection (Table S4). However, the role of JA pathway in susceptibility and tolerance FO is still controversial [39,40]. The emerged response to FOP infection in our study confirms the involvement of ET/JA-dependent pathways together with the activation of TIFY, ET-responsive TFs against FOP infection. Similar response has been observed by Sebastiani et al. [9] in melon against FO infection. Our data suggests that FOP infection activates the transcription of auxin related genes exclusively in FO-24 roots which in turn increases the auxin biosynthesis and indicates direct involvement of auxin in common bean-FOP pathosystem. Together with signaling and structural defense responses, our results envisage that common bean employs a cascade of defense mechanisms including structural and signaling responses and that the auxin, ET, and JA are the main hormones involved in common bean-FOP pathosystem similar to what has been reported in tomato and banana [9,18,22].

### 3.4. FOP Induced Energy Metabolism and Nitrogen Mobilization

Obligate biotrophs depend on host metabolism for intake of nutrients, which is a measure of pathogenicity of the fungus within the host. Many plant defensive compounds are derived from amino acid precursors such as glucosinolates and secondary metabolites [41,42]. Our results confirm that in common bean roots infected with FOP, amino acids and their derivatives such as N-acetlyl-L-tyrosin, L-cystathionin, glutathione oxidized, glutathione reduced form, 5-aminovaleric acid, and Nα-Acetyl-L-arginine were upregulated play a crucial role in defense against FOP. In relation to this, it is important to relate the significantly enriched pathway observed in KEGG enrichment scatter plot i.e., amino sugar and nucleotide sugar metabolism (Figure 3b). This reveals that primary metabolites i.e., amino acids and sugars are playing a critical role in innate defense pathways. The activation and rapid accumulation of amino acids and sugars affect FOP susceptibility as observed in chickpea [12]. The upregulation of glutamate synthase, glutamate dehydrogenase, and aspargine synthase indicate the role of nitrogen mobilization. Significant upregulation of these genes correlated well with metabolite outcome (Tables S4 and S5).

### 3.5. FOP Resistance in Common Bean is Mediated by Flavonoid Biosynthesis Pathway

It has been established that plants respond to pathogens by increased activation of the phenylpropanoid pathway leading towards biosynthesis of flavonoids, isoflavonoids, and phenolics. In our metabolome, the most significantly enriched pathway was flavonoid biosynthesis pathway (Figure 7). These compounds are regulated by chalcone synthase (CHS), chalcone isomerase (CHI), isoflavone synthase (IFS), isoflavone reductase (IFR), flavanone 3-hydroxylase (F3H), dihydroflavonol 4-reductase (DFR), anthocyanidin synthase (ANS), and leucoanthocyanidin reductase (LAR) genes and manipulated by MYB transcription factors [43]. As detailed in Table S5, these genes (except LAR gene(s)) were upregulated in FO-24 infected common bean roots. Similar results were revealed in chickpea infected with FO [12]. Previously, it was also known that fungal extract can induce these genes (CHI, IFS, and IFR) in Medicago cell suspension culture [44]. Consistently the accumulation of polyphenols, anthocyanins, flavanones, flavones, flavonoids, isoflavones, phenolamides, quinones, and terpenes identified by the metabolome profiles of CK-24 vs. FO-24 infected roots in the present study correlated well with transcriptomic data. Hence, the accumulation of flavonoid biosynthesis related genes and metabolites in FO-24 infected roots suggested their potential involvement in FOP resistance.

## 4. Materials and Methods

### 4.1. Plant Growth and In Vivo Inoculations

Seeds of common bean (Liyun No. 2) were obtained from the Lishui Institute of Agricultural Sciences, China. Seeds were sown in 15 cm diameter pots. The growing material filled in pots was sterile vermiculite and clay mixed in a 3:1 volume/volume ratio. The seedlings were allowed to grow under normal conditions i.e., 25 °C/18 °C day/night temperatures with a 16-h light/8-h dark photoperiod, and 60% humidity for 5 days. Plants were separated into two groups: control (CK) and *Fusarium oxysporum* f. sp. *phaseoli* (FOP) treated plants. Five individual seedlings were monitored at 4, 8, 12, and 24 h post infection. Then, 5-day-old seedlings were used for the infection at the fully expanded trifoliate leaves. The inoculum was prepared as reported earlier [3]. The control plants were supplemented with sterile ultrapure water. All the treated and control plants were evaluated for root quantitative traits such as root length, root volume, and fresh weight. As 24 h time point provided the best contrasting phenotype between CK and FO treated plants (Table S1, Figure 1), whole root samples from three individuals (biological replicates) in CK-24 and FO-24 were used for transcriptome and metabolome analysis. All treatments were grown in the same greenhouse with a 16 h light and 8 h dark cycle.

### 4.2. RNA Extraction, cDNA Library Construction, and Transcriptome Sequencing

Total RNAs were extracted using Spin Column Plant total RNA Purification Kit following the manufacturer′s protocol (Sangon Biotech, Shanghai, China) [45]. Purity of the extracted RNAs was assessed on 1% agarose gels as well as by NanoPhotometer spectrophotometer (IMPLEN, Los Angeles, CA, USA). For RNA quantification we used a Qubit RNA Assay Kit in Qubit 2.0 Flurometer (Life Technologies, Carlsbad, CA, USA). Further, RNA integrity was assessed by the RNA Nano 6000 Assay Kit of the Agilent Bioanalyzer 2100 system (Agilent Technologies, Santa Clara, CA, USA).

Sequencing libraries was created using NEB Next Ultra RNA Library Prep Kit following manufacturer′s instructions. The index codes were added to each sample. Briefly, the mRNA was purified from 3 μg total RNA of each of three replicate using poly-T oligo-attached magnetic beads. Subsequently, the fragmentation buffer was used to break the RNA into short fragments, and the short-fragment RNA was used as a template to synthesize the first strand cDNA with random hexamers, followed by buffer, dNTPs (dUTP, dATP, dGTP, and dCTP). The double-stranded cDNA was synthesized with DNA polymerase I, and then the double-stranded cDNA was purified using AMPure XP beads. The purified double-stranded cDNA was subjected to terminal repair, A tail was added, and the sequencing linker was ligated, and then AMPure XP beads were used for fragment size selection, and finally PCR enrichment was performed to obtain a final cDNA library. Library quality was initially quantified using Qubit 2.0 using the 2100 to test the insert size of the library followed by accurately quantifying the effective concentration of the library (>2 nM) by Q-PCR. Finally, six paired-end cDNA libraries with an insert size of 300 bp were constructed for transcriptome sequencing and sequenced on Illumina HiSeq platform (Illumina Inc., San Diego, CA, USA) by Wuhan MetWare Biotechnology Co., Ltd. (www.metware.cn).

### 4.3. De Novo Assembly, Functional Annotation, Classification, and Metabolic Pathway Analysis

The clean reads were retrieved after trimming adapter sequences, removal of low quality (containing > 50% bases with a Phred quality score < 20) and reads with unknown nucleotides (more than 1% ambiguous residues N) using the FastQC tool (http://www.bioinformatics.babraham.ac.uk/projects/fastqc/). GC content distribution check was performed. To stitch clean reads, Trinity was used (Version r20140717, [46]). For hierarchical clustering, Corset was used (https://code.google.com/p/corset-project/). The longest cluster sequence was obtained by clustering with Corset hierarchy as Unigene for subsequent analysis. The assembled unigenes were then aligned with various databases such as KEGG [47], GO [48], Clusters of Orthologous Groups (COG) [49], PfAM, Swissprot [50], egNOG [51], NR [52], KOG [53] using BLAST [54] with a threshold of E-value < 1.0 × 10^−5^.

The software KOBAS2.0 [55] was employed to get the unigene KEGG orthology. The analogs of the unigene amino acid sequences were searched against the Pfam database [56] using HMMER tool [57] with a threshold of E-value < 1.0 × 10^−10^. The sequenced reads were compared with the unigene library using Bowtie [58], and the level of expression was estimated in combination with RSEM [59]. The gene expression level was determined according to the FPKM.

### 4.4. Differential Expression and Enrichment Analysis

The read count was normalized and EdgeR Bioconductor package [60] was used to determine the differential expression genes (DEGs) between CK-24 and FO-24 with the fold change > 2 [61] and FDR correction set at *p* < 0.01. GO enrichment analysis was performed using the topGO method based on the wallenius noncentral hypergeometric distribution with *p* < 0.05 [62]. KEGG pathway enrichment analysis of the DEGs was done using KOBAS2.0 [55]. The FDR correction was employed (*p* < 0.05) to reduce false positive prediction of enriched GO terms and KEGG pathways.

### 4.5. Quantitative RT-PCR Analysis

Eight DEGs, characterized by interesting expression profiles in response to FOP infection in FO-24 common bean plants were selected for qRT-PCR. First strand cDNAs were synthesized from 100 ng of total RNA using the High Capacity cDNA Reverse Transcription Kit (Applied Biosystem, Carlsbad, CA, USA). Primers were designed using Primer3 Software (http://frodo.wi.mit.edu/primer3/; Table S7) and the specificity was checked by blasting their sequences in the NCBI database. The *Actin* constitutively expressed gene was used as the reference gene [3]. All qRT-PCR reactions were carried out on a Rotor-Gene 6000 machine (Qiagen, Shanghai, China) with the following thermal cycling profile: 50 °C for 2 min and 95 °C for 2 min, followed by 40 cycles at 95 °C for 3 s and 60 °C for 30 s. Melting curve analysis was performed to verify single product amplification with temperature ranging from 55 to 95 °C by increasing of 1 °C every step. All reactions were performed in a total volume of 10 μL containing 30 ng of cDNA, 5 μL 1 × SYBR^®^ Select Master Mix (Applied Biosystem, Carlsbad, CA, USA), and 0.2 μL (20 μM) of each primer. For each sample, two biological replicates were analyzed in independent runs and a no-template control was included for each gene. Intra-assay variation was evaluated by performing all reactions in triplicate. The quantification cycle (Cq) was automatically determined using Rotor-Gene 6000 Series Software, version 1.7 as reported earlier [9].

### 4.6. Widely Targeted Metabolomics

The sample preparation, extract analysis, metabolite identification, and quantification were performed at Wuhan MetWare Biotechnology Co., Ltd. (www.metware.cn) following their standard procedures [30].

### 4.7. Sample Preparation 

The vacuum freeze-dried root samples were crushed using a grinder (MM 400, Retsch, Haan, Germany) to a powder. A total of 100 mg powder was weighed and aliquots were extracted at 4 °C with 0.6 mL 70% aqueous methanol and vortexed six times to increase the extraction rate. After centrifuging at 10,000× *g* for 10 min, the supernatant was aspirated, and the sample was filtered through a microporous membrane (0.22 μm pore size) and stored in a sample bottle for UPLC-MS/MS analysis.

### 4.8. Chromatographic Mass Spectrometry Acquisition Conditions

The data acquisition instrument system included Ultra Performance Liquid Chromatography (UPLC) (Shim-pack UFLC SHIMADZU CBM30A, https://www.shimadzu.com.cn/) and tandem mass spectrometry (SHIMADZU Corp., Kyoto, Japan) (MS/MS) (Applied Biosystems 4500 QTRAP, http://www.appliedbiosystems.com.cn/). The liquid phase conditions included (1) column: waters ACQUITY UPLC HSS T3 C18 1.8 μm, 2.1 mm × 100 mm; (2) mobile phase: phase A = ultrapure water (0.04% acetic acid was added), phase B = acetonitrile (0.04% acetic acid was added); (3) elution gradient: 0.00 min B = 5% in comparison, B was linearly increased to 95% in 10.00 min, and maintained at 95% 1 min, 11.00–11.10 min, B was reduced to 5%, and was 5% balanced to 14 min; (4) flow rate 0.35 mL/min; column temperature 40 °C; injection volume 4 μL. Whereas the mass spectrometry conditions were as following: the electrospray ionization (ESI) temperature was 550 °C, the mass spectrometry voltage was 5500 V, the curtain gas (CUR) was 30 psi, and the collision-induced dissociation (CAD) parameter was set high. In the triple quadrupole (QQQ), each ion pair was scanned for detection based on optimized decolusting potential (DP) and collision energy (CE) [63].

Based on the self-built database MWDB (metware database) at Wuhan MetWare Biotechnology Co., Ltd. (www.metware.cn), the material was characterized according to the secondary spectrum information. The isotope signal was removed during the analysis, and the repeated signals including K+ ions, Na+ ions, NH4+ ions, and fragment ions which are themselves other larger molecular weight substances were removed.

Metabolite quantification was performed using multiple reaction monitoring (MRM, as shown below) in triple quadrupole mass spectrometry. In the MRM mode, the fourth-stage rod first screens the precursor ions (parent ions) of the target substance, and excludes the ions corresponding to other molecular weight substances to initially eliminate the interference; the precursor ions break through the collision chamber to induce ionization and break to form a lot of fragment ions. The triple quadrupole filter is then used to select a desired feature fragment ion to eliminate non target ion interference, which makes the quantification more accurate and repeatable. After obtaining metabolite mass spectrometry data for different samples, peak area integration was performed on the mass spectral peaks of all the substances, and the mass spectral peaks of the same metabolite in different samples were integrated [64].

### 4.9. Metabolomics Data Analysis 

Data matrices with the intensity of metabolite features under FOP and control conditions were uploaded to the Analyst 1.6.1 software (AB SCIEX, Ontario, Canada). For statistical analysis, missing values were assumed to be below the limits of detection, and these values were imputed with a minimum compound value [63]. The relative abundance of each metabolite was log transformed before analysis to meet normality. A Dunnett’s test was used to compare the abundance of each metabolite between control and FOP. False discovery rate was used for controlling multiple testing. The supervised multivariate method, partial least squares-discriminant analysis (PLS-DA), was used to maximize the metabolome difference between the control and FOP treated samples. The relative importance of each metabolite to the PLS-DA model was checked using a parameter called the variable importance in projection (VIP). Metabolites with VIP > 1.0 were considered as differential metabolites for group discrimination. Principal Component Analysis (PCA), Hierarchical Cluster Analysis (HCA), and KEGG pathway analysis were performed in R software (www.r-project.org).

## 5. Conclusions

In the present study the whole transcriptome and metabolome of common bean infected by FOP 24 hpi were characterized. The differences in terms of DEGs between the inoculated and non-inoculated common bean showed that nitrogen metabolism and energy metabolism participated in defense response to FOP infection. Flavonoid pathway was the main defense response in common bean. Transcriptome analysis showed that the spliceosome, RNA transport, ribosome biogenesis in eukaryotes, proteasome, and phenylalanine metabolism were the top five significantly enriched pathways. Cell-wall related genes proved to be the first response to FOP attack and started a cascade of signaling leading to accumulation of cell wall degradation related transcripts. PAMP/MAMP, PRRs, and PRs were being regulated in response to FOP infection suggesting triggering of immunity in common bean. Activation of systematic acquired resistance was also observed in our study where the role of hormones in the signaling pathway was observed. These results demonstrate the common bean in response to FOP utilizes different and effective defense pathways comprising of a complex resistance network of structural, signaling, and chemical responses. Further investigations will be focused on functional validation and mapping of the DEGs, which could represent a helpful tool for developing common bean resistant varieties toward FOP.

## Data Availability

The data for RNA-sequencing is available at National Center for Biotechnology Information (NCBI) with accession number PRJNA589754.

## Supplementary Materials

Supplementary files can be found at https://www.mdpi.com/1422-0067/20/24/6278/s1. Supplementary Table S1. Comparison of means for root quantitative data. Supplementary Table S2. Summary of sequencing output statistics. Supplementary Table S3. Mapping results of RNA-Seq data from inoculated and non-inoculated common bean seedlings. Supplementary Table S4. Complete list of differentially expressed genes regulated by infection of FOP at 24 hpi in control and infected roots. Supplementary Table S5. List of all detected metabolites. Supplementary Table S6. List of differentially expressed metabolites. Supplementary Table S7. List of primers used for qRT-PCR analysis. Supplementary Figure S1. Differential expression gene function annotation and enrichment analysis. Supplementary Figure S2. Orthogonal partial least squares discriminant analysis.
