# Supplementary material for: Combined De Novo Transcriptome and Metabolome Analysis of Common Bean Response to Fusarium oxysporum f. sp. phaseoli Infection
Source: Int J Mol Sci. 2019 Dec 12;20(24):6278. doi: 10.3390/ijms20246278 (PMC6941151; doi:10.3390/ijms20246278)
Supplement: Supplementary file 1 [file ijms-20-06278-s001.zip › ijms-663480-SI-Author Proofreading/ijms-663480-SI-Author Proofreading.docx]

Supplementary Figure S1. Differential expression gene function annotation and enrichment analysis


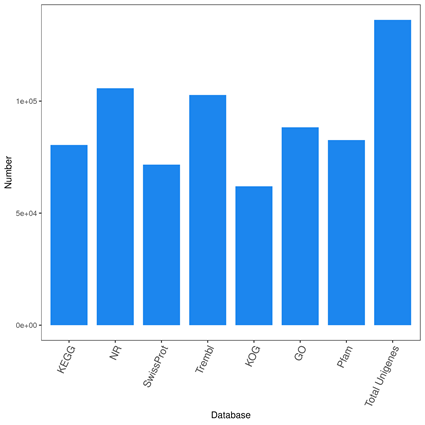


**Figure S1 A.** Unigene annotated chart.


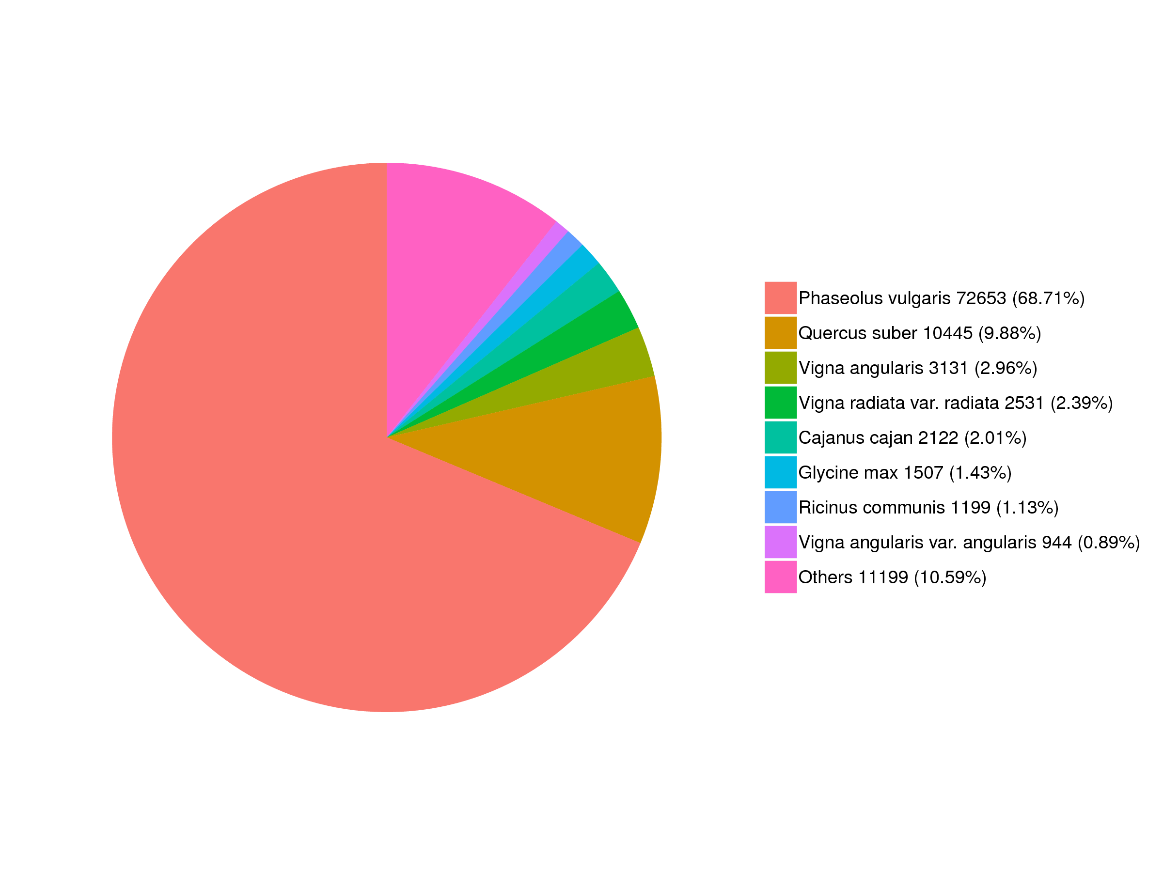


**Figure S1 B.** NR database annotation statistics. Similarity of the transcript sequence of common bean with similar species.


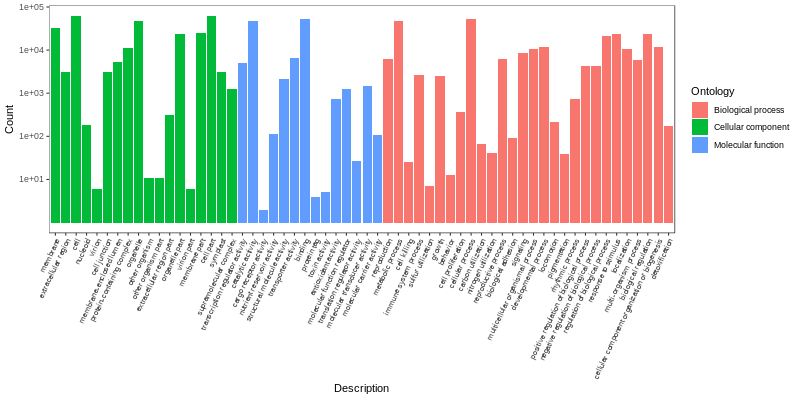


**Figure S1 C.** GO classification histogram. Successfully annotated genes are classified according to the GO biological process (BP), cellular component (CC), and molecular function (MF).


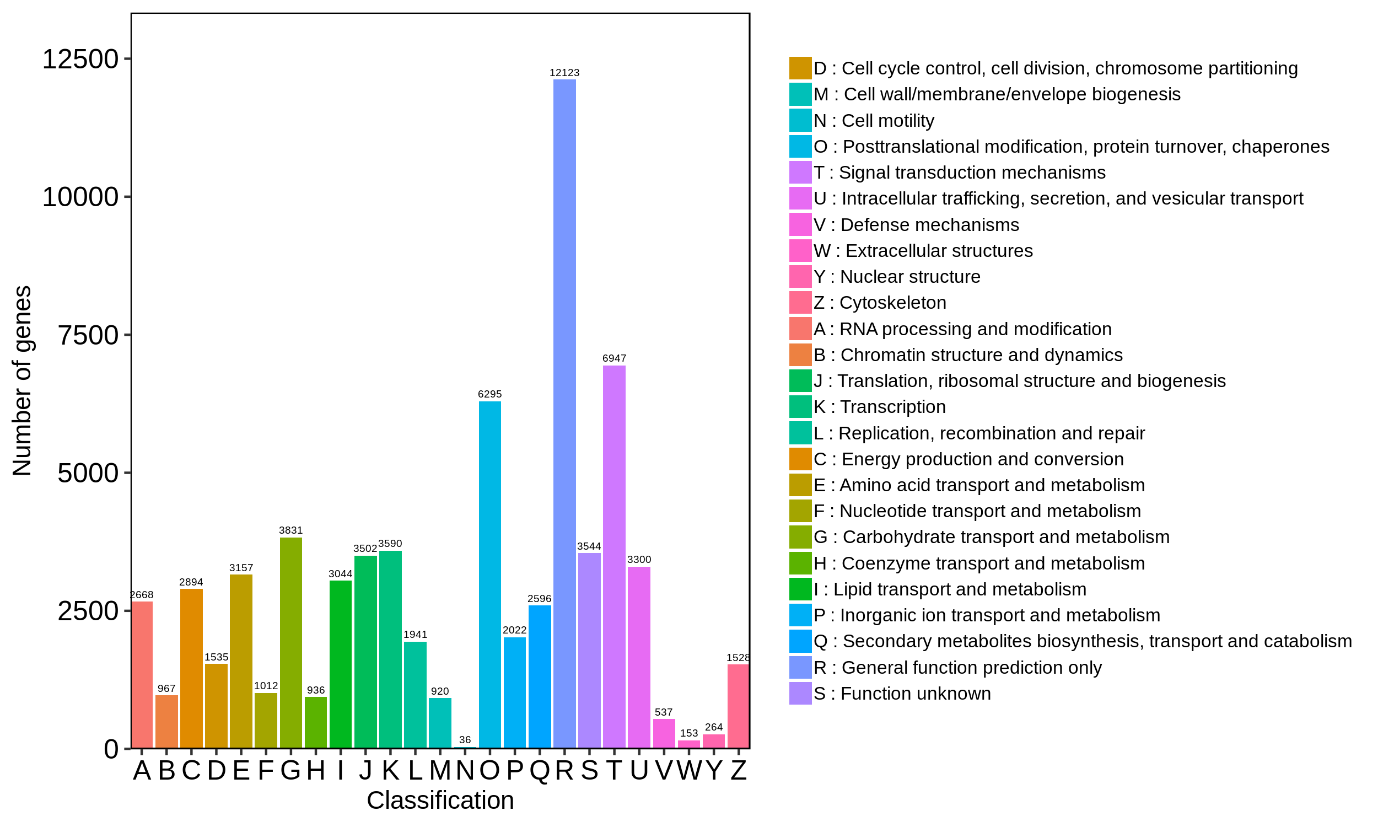


**Figure S1 D.** KOG classification of unigenes.

Figure S2. Orthogonal partial least squares discriminant analysis


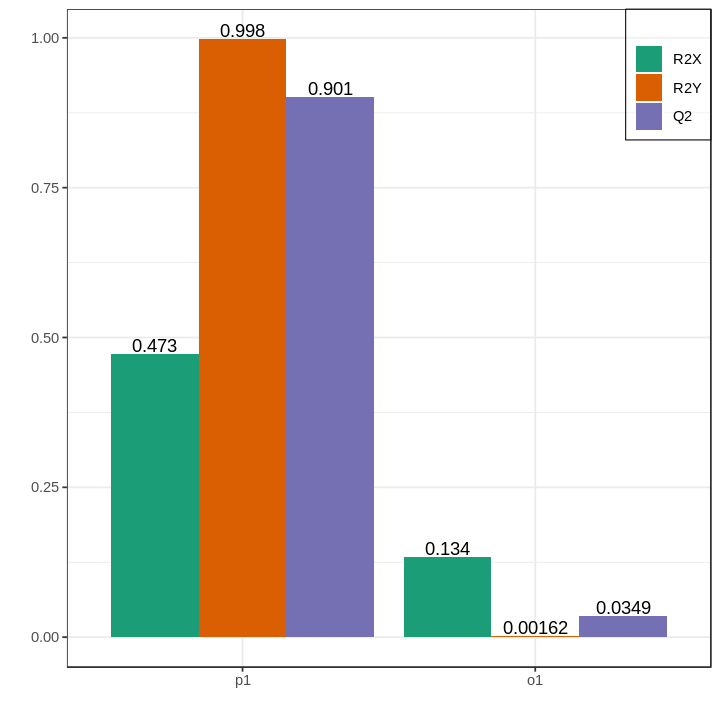


**Figure S2 A.** OPLS-DA model summary: in the abscissa, p1 represents the predicted principal component, o1 represents the orthogonal principal component, and the ordinate represents the corresponding R2X, R2Y, and Q2.


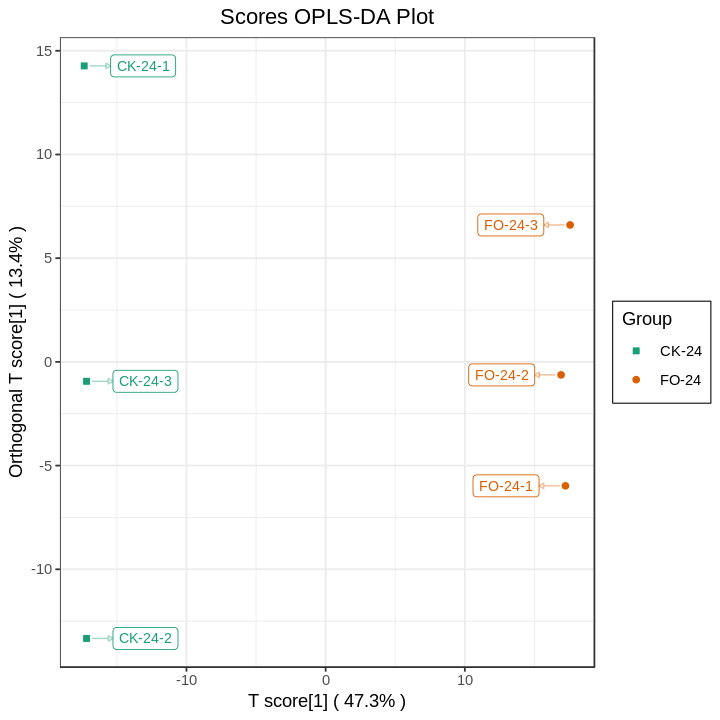


**Figure S2 B.** OPLS-DA S-plot: the abscissa indicates the covariance of the principal component and the metabolite, and the ordinate indicates the correlation coefficient between the principal component and the metabolite. The closer to the upper right and lower left corners, the metabolites indicate that the difference is more significant, red dots indicate that these metabolites have a VIP value greater than or equal to 1, and green dots indicate that these metabolites have a VIP value of less than one.


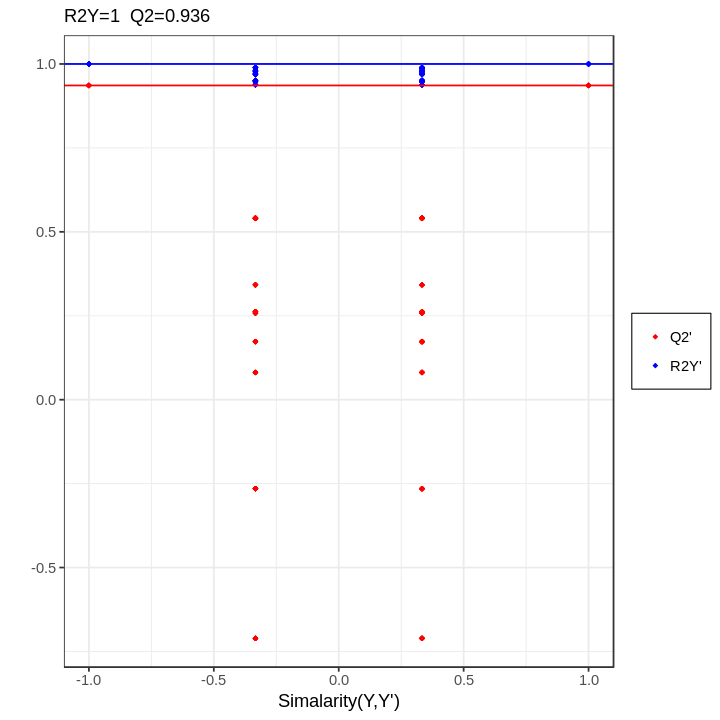


**Figure S2 C.** OPLS-DA verification chart: the alignment of OPLS-DA was performed (*n* = 200, that is, 200 alignment experiments were performed). In the model verification, the horizontal line corresponds to R2Y and Q2 of the original model, and the red and blue points represent R2Y' and Q2' of the model after Y replacement, respectively. If both R2Y' and Q2' are smaller than R2Y and Q2 of the original model, that is, the corresponding points do not exceed the corresponding line, then the model is meaningful, and the differential metabolites can be screened according to the VIP value analysis.


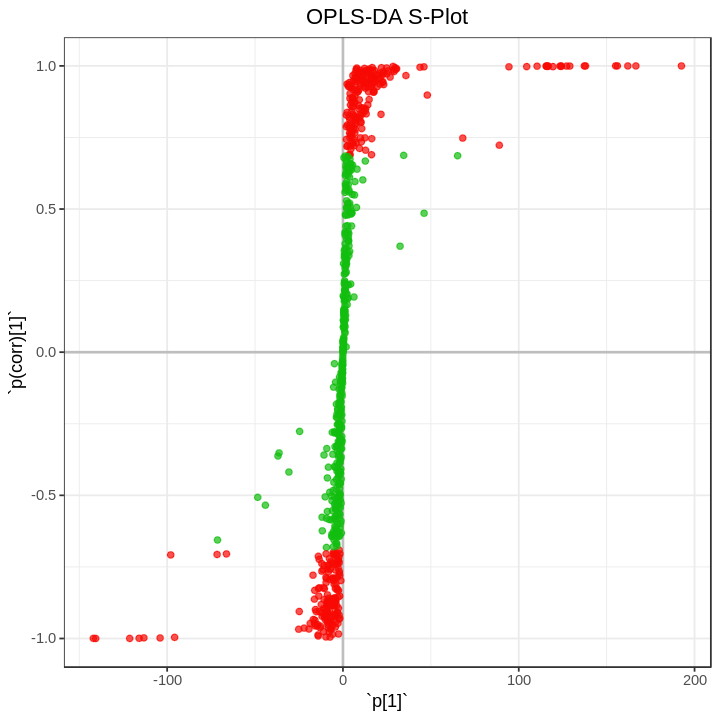


**Figure S2 D.** OPLS-DA score chart.
